# The Usefulness of Magnetic Resonance Angiography to Analyze the Variable Arterial Facial Anatomy in an Effort to Reduce Filler-Associated Blindness: Anatomical Study and Visualization Through an Augmented Reality Application

**DOI:** 10.1093/asjof/ojab018

**Published:** 2021-05-11

**Authors:** Marc Mespreuve, Karl Waked, Barbara Collard, Joris De Ranter, Francis Vanneste, Benoit Hendrickx

**Affiliations:** Department of Medical Imaging, University Hospital Ghent, Ghent, Belgium; Department of Plastic and Reconstructive Surgery, University Hospital Brussel, Brussel, Belgium; AZ Zeno Hospital, Knokke, Belgium; Department of Plastic and Reconstructive Surgery, Universitair Ziekenhuis Leuven Campus Gasthuisberg, Leuven, Belgium; Department of Medical Imaging, AZ Zeno Hospital, Knokke, Belgium; Department of Plastic and Reconstructive Surgery, University Hospital Brussel, Brussel, Belgium

## Abstract

**Background:**

The use of soft tissue fillers for facial rejuvenation is increasing rapidly and the complications, unfortunately, follow the same path. Blindness caused by intravascular filler injections is a rare but devastating complication. Knowledge of the individual arterial anatomy may aid the injector in avoiding injecting into an artery and thus to prevent blindness.

**Objectives:**

To evaluate if the use of magnetic resonance angiography (MRA) may visualize the arterial facial anatomy in a contrast- and radiation-free way and study the individual arterial variations using an augmented reality (AR) image.

**Methods:**

The individual arterial anatomy of the 3 terminal branches of the ophthalmic artery (supraorbital [SO]; supratrochlear [STr]; and dorsal nasal [DN] arteries) of 20 volunteers was studied by a 3-Tesla MRI, combining infrared (IR) facial warming and 3-dimensional time-of-flight multiple overlapping thin slab acquisition MRA. The resulting visualization of the facial arteries was shown on the patient’s face through AR technology.

**Results:**

The MRA was able to visualize the SO in 90.0%, STr in 92.5%, and DN arteries in 75% of the examined patients, as well as numerous variations in both vessel localization and path. Furthermore, a proof-of-concept of the AR visualization of the individual arterial anatomy was successfully implemented.

**Conclusions:**

Dermal filler injectors should be aware of the risk of filler-induced blindness and familiarize themselves with the visualization of the variable facial vascular anatomy. The implementation of a one-time MRA and subsequent AR visualization may be useful in the accurate planning of minimally invasive facial rejuvenation procedures.

The incremental use of soft tissue fillers (STF) for facial rejuvenation—mostly hyaluronic acid (HA)—has led to an increasing number of adverse reactions reported in literature.^1-4^ In spite of the potential reversibility of intra-arterial HA injections by use of hyaluronidase (which has the ability to dissolve HA), occlusion of the central retinal artery and subsequent blindness may still occur.^5,6^ Even in the hands of experienced injectors who adhere to the safety procedures and possess the knowledge on the (standard) arterial anatomy, intra-arterial injection of STF in one of the 3 main terminal facial branches of the ophthalmic artery—supratrochlear (Str), supraorbital (SO), and/or dorsal nasal (DN) artery—remains an unforeseen complication and may cause retrograde embolization of the ophthalmic artery. Subsequent occlusion of the central retinal artery with secondary retinal ischemia may in turn lead to blindness.^7-13^ Knowledge on the complex anatomy of the facial arteries is of course essential.^14^ However, the differences between the *textbook* “straight” and *real-life* “tortuous” arterial course, as well as—and more importantly—the individual anatomical variability with regards to arterial depth and location explain the high risk associated with intravascular STF injections.^15^

A 3-dimensional time-of-flight multiple overlapping thin slab acquisition (3D-TOF MOTSA) magnetic resonance angiography (MRA), a harmless and radiation-free visualization method of the facial and ophthalmic arteries and their superficial branches, seems to be a very useful method in analyzing the individual arterial map of the patient’s face.^16-18^ It may therefore be of use to better plan dermal filler injections, as it allows the injector to take into account the individual (and variable) localization of the arteries around the eye.

## METHODS

Between June and August 2020, a total of 20 volunteers were recruited. The only inclusion criterion was age (between 18 and 65 years). Exclusion criteria were non-MRI-compatible internal devices (such as a pacemaker); metal plates in the face or skull; dental braces (but not dental wires); tattoos on the face (but not permanent makeup); claustrophobia; vascular disease; and congenital or acquired facial anomaly. The purpose of the study and the examination technique was explained to all volunteers and their informed consent was obtained before the MRA examination after being completely and thoroughly informed regarding the purpose of the study, as well as the planned procedure and its potential risks. This study was approved by the ethical board of the hospital (AZ Zeno Knokke/EC 2019-392) and was fully conducted in the hospital of AZ Zeno in Knokke (Belgium). All procedures performed in studies involving human participants were in accordance with the ethical standards of the institutional and/or national research committee and with the 1964 Helsinki Declaration and its later amendments or comparable ethical standards.

All images were acquired on a 3-Tesla (3-T) full-body MR system (Signa Pioneer, General Electric, Boston, MA), using a dedicated 21-channel head coil. Before the TOF MRA examinations, all volunteers were positioned with closed eyes in front of an infrared (IR) light source (Philips PR 3120 of 300 W [Eindhoven, The Netherlands] with an Infracare screen, which filters out the UV light) at a distance of 30 cm and with their face parallel to the lamp for 10 minutes. The heath induced vasodilatation and enhanced the vascular flow, both aiding in an enhanced image acquisition.^17,18^ At the same time, the patients were asked to stimulate their facial muscles by slowly moving their lips and forehead and switching between several facial expressions during the exposure time in order to further enhance the visualization of the facial arteries (by vascular dilatation and increased flow speed).^19^ After the acquisition of the scout views, a 3D-TOF MOTSA MRA sequence was acquired in an oblique coronal plane (tilting of 25° backwards vs the line between the glabella and the chin). The MRA protocol is summarized in Table 1 and has been developed, based on a previously published study discussing the MRA sequence in more detail.^17^ The acquisition time was 13 minutes and 30 seconds. During that time, the patient was asked to remain completely still with the eyes and mouth closed and the face parallel to the examination table. A multislab technique was used to reduce the saturation effect of the inflowing blood signal. Maximum Intensity Projection (MIP) images were made every 2° over 180° in a sagittal plane from right to left. The attained voxel size of the acquired MRI images was 0.4 × 0.4 × 0.5 mm^3^, allowing to visualize vessels smaller than 1 mm in diameter.

**Table 1.** Three-Dimensional Fast Spoiled Gradient-Echo Sequence^a^

TR	30 ms
TE	6.8 ms
Acquisitions	5
FOV	180 mm
Flip angle	30°
Matrix (frequency/phase)	256 × 240 pixels
Slice thickness	0.5 mm
Averages	1
SNR	1.0
Voxel size	0.7 × 0.8 × 0.5 mm
Time of acquisition	13 min 30 s

FOV, field of view; SNR, signal-to-noise ratio; TE, time of echo; TR, time of repetition. ^a^Gradient-echo sequence with 7 overlapping (17.5%) slabs.

The source and MIP images were randomized and independently assessed by an experienced radiologist (M.M.; 33 years of experience) and a plastic surgeon (B.H.; 16 years of experience)—both familiar with the facial anatomy—in order to evaluate the successful visualization of the 3 aforementioned facial vessels (SO, ST, and DN arteries) as well as to analyze potential differences in the individual location and course of these 3 terminal branches of the ophthalmic artery. The location of the arteries was compared to the known anatomical study data of Koziej et al.^20^ Fleiss' kappa was used to evaluate the interrater agreement.

The images were displayed on a medical monitor, 6 Megapixel Barco display, 32″, 3280 × 2048 resolution (Barco/Kortrijk, Belgium).

## RESULTS

Of the 20 volunteers, there were 5 men (25%) and 15 women (75%). The age ranged from 22 to 62 years, with a mean age of 43.3 ± 4.6 years. All 20 participants completed the whole MRA protocol successfully. In spite of the examination time of 13 minutes 30 seconds on a 3-T MRI, we did not notice significant motion artifacts. The low mean age and the motivation of these patients seemed to encourage their cooperation. Although the reaction of the skin (hyperemia) to the IR exposure was visible, no adverse reactions due to the “IR enhancement” were noted, nor mentioned. The warmth sensation remained for an average of 2 hours. A total of 40 (20 right sided and 20 left sided) small and/or tortuous DN, SO, and STr arteries were evaluated for their successful visualization (Table 2) and, if visible, their differences in position and course.

**Table 2.** Individual Visual Scores for the Main Trunk of the SO, STr, and DN Arteries (Right and Left)

	Right			Left		
P	SO	STr	DN	SO	STr	DN
1	**+**	**+**	**+**	**+**	**+**	**+**
2	**+**	**+**	**+**	**+**	**+**	**+**
3	**+**	**+**	**+**	**+**	**+**	**+**
4	**+**	**+**	**+**	**+**	**+**	**+**
5	**+**	**+**	**+**	**+**	**−**	**+**
6	**+**	**+**	**+**	**+**	**+**	**+**
7	**+**	**+**	**+**	**+**	**+**	**+**
8	**+**	**+**	**−**	**+**	**+**	**−**
9	**−**	**−**	**+**	**−**	**−**	**+**
10	**+**	**+**	**−**	**+**	**+**	**−**
11	**+**	**+**	**+**	**+**	**+**	**+**
12	**+**	**+**	**+**	**+**	**+**	**+**
13	**+**	**+**	**−**	**+**	**+**	**−**
14	**+**	**+**	**+**	**+**	**+**	**−**
15	**+**	**+**	**+**	**+**	**+**	**+**
16	**+**	**+**	**+**	**+**	**+**	**+**
17	**+**	**+**	**+**	**+**	**+**	**−**
18	**−**	**+**	**−**	**+**	**+**	**+**
19	**−**	**+**	**+**	**+**	**+**	**−**
20	**+**	**+**	**+**	**+**	**+**	**+**

DN, dorsal nasal; P, participant; SO, supraorbital; STr, supratrochlear. **−** = not visualized; **+** = visualized.

**Figure 1.**
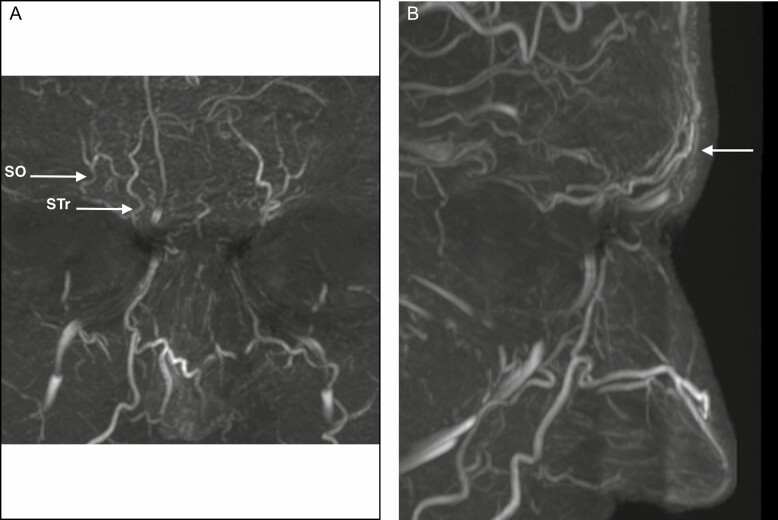
Supratrochlear (STr) and supraorbital (SO) arteries in a frontal and profile view of a 32-year-old male. (A) Frontal view with SO and Str arteries; and (B) profile view showing the superficial location of both arteries.

**Figure 2.**
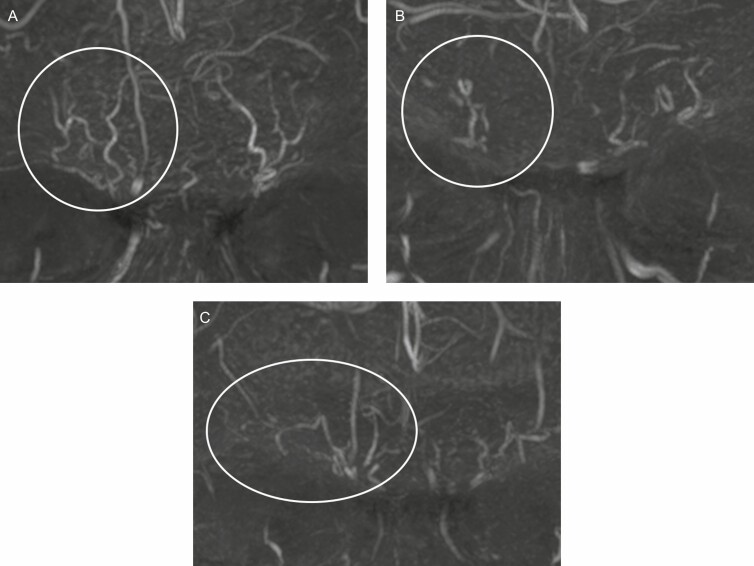
Variability of the supratrochlear (STr) and supraorbital (SO) arteries in a frontal view. (A) Parallel tortuous course at a distance (7 right and 3 left sided) in a 32-year-old male. (B) Close tortuous course (6 right and 9 left sided) in a 41-year-old male. (C) A parallel tortuous course or a close tortuous course with branching connection of the SO to the temporal region (4 right and 3 left sided) in a 50-year-old female.

The STr artery (Figures 1 and 2A) is a small, tortuous vessel running paramedian and ascending bilaterally just above the superomedial border of the orbit. Starting in a subperiosteal plane, the STr artery becomes very quickly superficial during its ascending course through the Corrugator and Frontalis muscles. It was observed in 92.5% of our cases (18/20 on the left side and 19/20 on the right side). The STr artery was consistently located in a 10-mm-wide area 1 cm lateral from the midline, which is in accordance with cadaver studies by postmortem computed tomography (CT) during forensic autopsy.^20^

The SO artery emerges more laterally, usually above the center of the orbit running almost parallel to the STr (Figures 1 and 2A), but at a variable distance from each other. It was observed in 90.0% of our cases (17/20 on the left side and 19/20 on the right side). Based on our pool of patients and previous studies, it should be expected in a slightly wider (11 mm) area 2 cm lateral from the midline or 11 mm from the medial canthus.^20,21^

**Figure 3.**
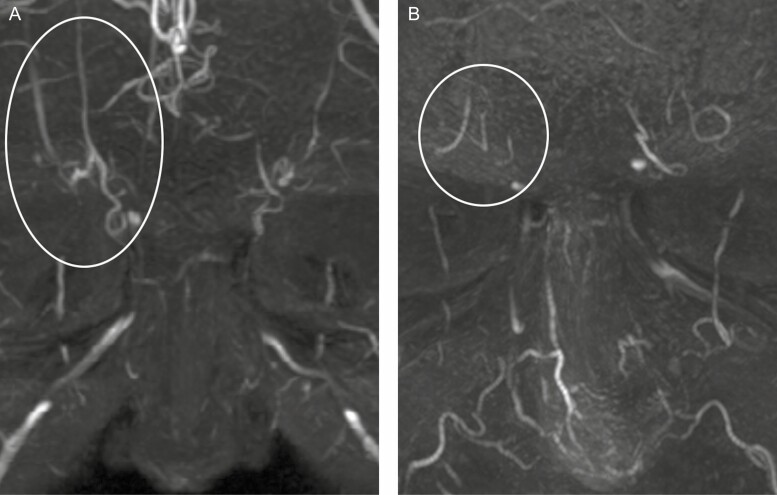
Variants of the supratrochlear (STr) and supraorbital (SO) arteries in a frontal view. (A) More rectilinear course of STr and SO (5 right and 4 left sided) in a 29-year-old female. (B) Very small STr and SO close to each other (1 right and 1 left sided) in a 62-year-old male.

As noticed, both vessels may also run closer to each other (Figure 2B) with a mere distance of only 4 mm between both arteries. It is well known that the SO artery often gives a lateral branch that anastomoses to the anterior branch of the superficial temporal artery, thus forming an important (and potentially hazardous) connection between the internal and external carotid system (Figure 2C). The course of both the STr and SO arteries may be rectilinear (Figure 3A) or more tortuous (Figure 2A). The caliber may also vary (Figure 3B shows both arteries with a small diameter). The STr artery may occasionally start in a far lower medial position (Figure 3A) than its expected topographic location (ie, on the line of the medical canthus). For the proximal main trunks of the STr and SO arteries, we did not observe an overlap between the zones of occurrence.^20^

**Figure 4.**
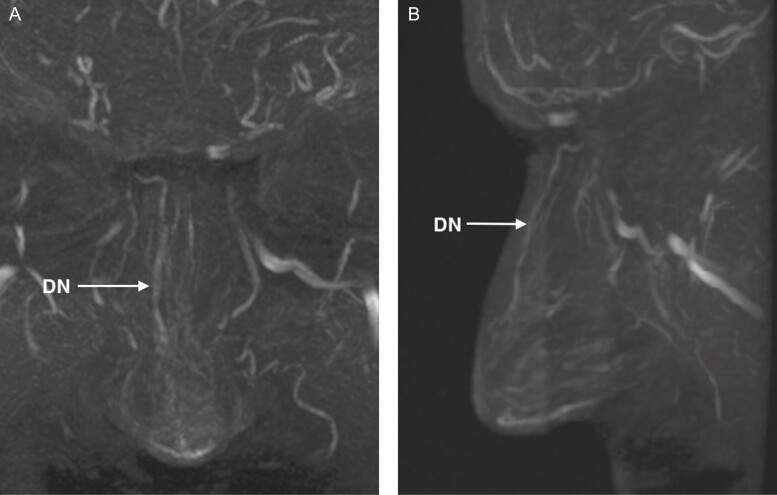
Dorsal nasal (DN) artery in a frontal and profile view in a 41-year-old male. (A) Frontal view showing complete course of the DN artery; and (B) profile view showing the subcutaneous course of the DN artery.

**Figure 5.**
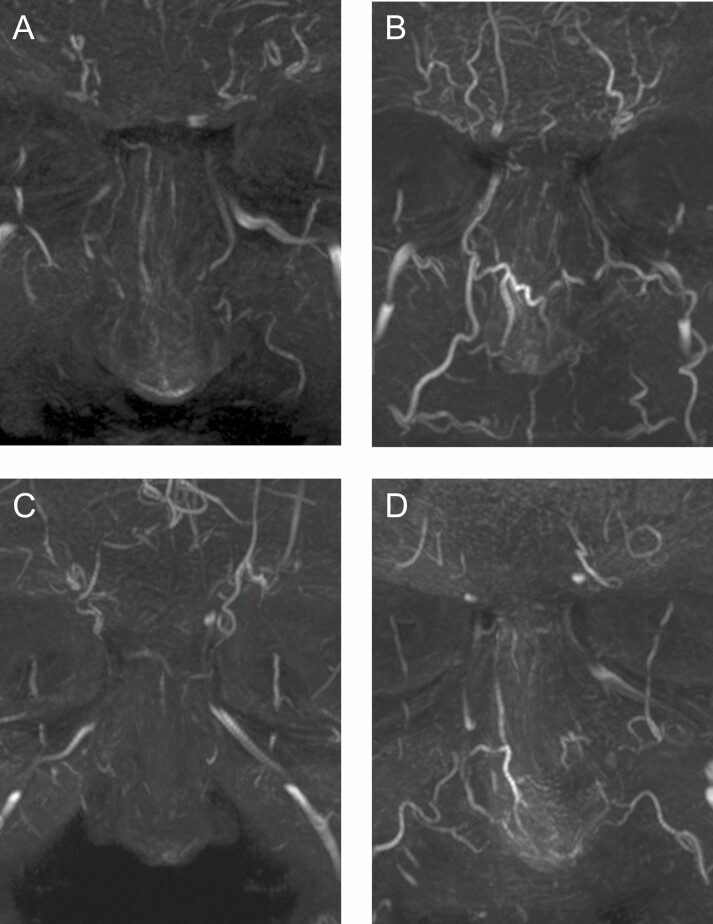
Variability of the dorsal nasal (DN) artery in a frontal view. (A) One side (right) dominates more or less the vascular supply (8 right and 10 left sided) in a 41-year-old male. (B) Only the distal part is markedly developed on both sides. Proximally only a network of small vessels (rete) is present in this 32-year-old male. (C) Global hypoplastic DN vessels in a 29-year-old female. (D) Proximally short bilateral DN, with a distal part in connection with the lateral nasal artery and connections over the midline in a 62-year-old male.

The DN artery was visualized in 75.0% of the cases (16/20 on the left side and 14/20 on the right side). Its localization and branching pattern may vary significantly (Figure 4). One side may be dominant and cross the midline (Figure 5A) or both DN arteries may vascularize the dorsum of the nose equally.^22,23^ Sometimes, only the distal part is markedly developed on both sides and more proximally only a network of small vessels (rete) may be present (Figure 5B). Global hypoplastic DN vessels may also occur (Figure 5C). A proximal short bilateral DN, with a distal part in connection with the lateral nasal artery and connections over the midline may be present as well (Figure 5D).

Fleiss' kappa for interrater agreement on identification and location for all the 3 end arteries of the ophthalmic artery was between 0.81 and 1.00 (almost perfect agreement).

Finally, we also noticed in 35% of the scanned patients the presence of a paracentral artery (Figure 1). This is not a new finding, as its existence has been discussed in previous publications.^20^ The paracentral artery originates as a continuation of the angular artery on the forehead or arises from the communicating branch with the supratrochlear artery. The paracentral artery was—as described previously and also visualized in this study—in a 13-mm-wide zone 2 mm lateral from the midline.^20^

## DISCUSSION

**Figure 6.**
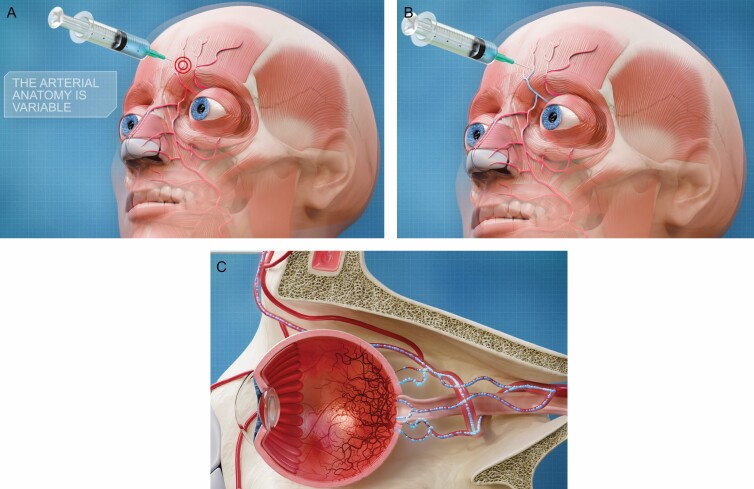
Pathway of an intra-arterial soft tissue filler (STF) injection causing blindness. (A) Accidental injection of a filler product in the variable location of (either) the supraorbital; supratrochlear; and/or dorsal nasal artery. (B) As the pressure of the filler syringe is higher than the blood pressure, the filler product is pushed in a retrograde direction toward the ophthalmic artery. (C) As soon as the pressure on the filler syringe is released, the filler product is transported in an antegrade direction through the central retinal and/or short and long posterior ciliary artery, causing ischemia of the retina.

Numerous papers elucidate on the multiple variations of the arterial anatomy of the face.^24-26^ Together with the rising number of STF injections and reported complications, this feeds the need to develop a (preferably harmless) imaging technique that allows any injector to know the individual vascular facial anatomy of their patients in order to locate, and potentially avoid, the arteries at risk during STF injections. Vision loss, total or partial, is a relatively rare complication but is of grave concern due to its disastrous outcome of which hundreds of cases have been described in literature.^27^ It may occur following filler injections in the nasal region or forehead (Figure 6A). A high pressure (greater than the sum of the systolic arterial pressure and the frictional forces due to viscous flow) retrograde injection (Figure 6B) of exceedingly small amounts (0.05-0.1 mL) of filler product into (one of) the 3 orbital branches (SO, STr, and DN artery) or connecting branches nearby (such as the anterior branch of the superficial temporal artery) will ultimately reach the ophthalmic artery where further antegrade flow (when the pressure on the plunger of the filler syringe is released) may obstruct the branches of the central retinal artery, causing retinal ischemia (Figure 6C).^5,28-30^

As filler injections are elective and (usually) aesthetic procedures, this imaging technique would preferably be noninvasive, radiation-free, and contrast-free. The technique we describe meets all these criteria and is therefore the best-suited technique to identify the vascular anatomy of the terminal branches of the ophthalmic artery prior to STF injections.

In MRA bright-blood methods, the signal from the moving protons is accentuated relative to the stationary protons of the surrounding tissue.^31,32^ These flow-based techniques include mainly time-of-flight (TOF), phase contrast (PC), and fast spin echo imaging (FSE).^33,34^ TOF is the most time-efficient method for obtaining MRA images.^35,36^

In this study, a 3D-TOF MOTSA MRA was performed in 20 volunteers. People aged 35 to 50 years accounted for 75% and the age group of 51 to 64 accounted for 25%. This correlates with the data from the cosmetic surgery national data bank statistics, showing that patients in their forties and fifties are the most common age group seeking non-surgical aesthetic procedures.^37^

Only one MRI sequence is needed to capture the superficial facial arteries (with a total duration of 13 minutes 30 seconds in case of a GE 3-Tesla MRI), together with an MIP reconstruction every 2° over 180° from right to left to visualize the MRA data.

### MRA Findings

We illustrated in a randomly selected group of volunteers already a very variable appearance and course of the small and/or tortuous DN, SO, and STr arteries in a mixture of males and females, younger and older patients.^28^ This image data analysis, as well as the pool of MRI data that may be collected in the future as more injectors may refer their patients for this type of MRA, further supports the description of “high-risk” and “low-risk” zones in the face, that is, zones that are at high risk for a vascular complication during filler injections vs safer zones. As MRI data are much easier to collect than anatomical data from cadaver dissections—most anatomical assumptions are currently based on cadaver studies—we may assume that more extensive anatomical studies will be feasible thanks to the ever-increasing pool of MRI scans that will be made once this sequence gets implemented in more hospitals.

### Visualizing the Anatomy Through Augmented Reality: A Glimpse of the Future

**Figure 7.**
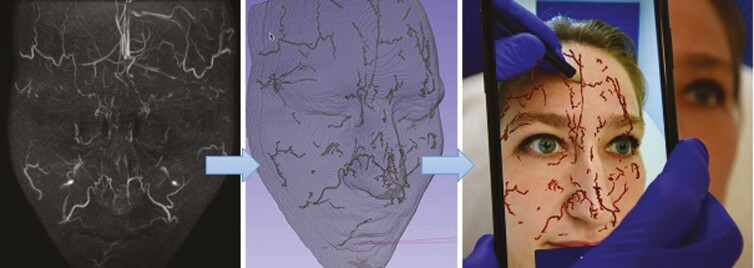
Magnetic resonance angiography findings and augmented reality (AR) visualization in a 26-year-old female. (A) Infrared thermally enhanced MRI without contrast. Frontal view of a maximum intensity projection (MIP) from the 3-dimensional time-of-flight multiple overlapping thin slab acquisition (3D-TOF MOTSA). (B) Segmentation of subcutaneous arteries. 3D image after isolation of the arteries and conversion to a 3D volume. (C) Visualization of subcutaneous arteries through AR and projection on the face of the patient with a smartphone camera.

In order to implement the developed MRA protocol in the injector’s daily practice, the acquired MRA images were used to develop an augmented reality (AR) image of the patient’s individual arterial network. In a proof-of-concept setting, the native DICOM images were processed by a 3D-software program which isolates the superficial, subcutaneous arteries of the face and creates a 3D volume of the arterial network. Dedicated facial recognition software was then implemented to visualize the arterial network of the patient through AR on the face of the patient by use of a smartphone (Figure 7). This AR application may aid the injector in avoiding plunging a filler needle right into one of the periorbital arteries.

In light of the increasing reports on complications secondary to intravascular injection (embolia medicamentosa) of a STF, the IR-MRA visualization of these arteries through AR may indeed help to prevent these life-changing adverse events. This is especially relevant in case of filler-associated blindness, as there is currently no clear and effective treatment protocol, despite the number of challenges that exist in trying to avoid an intra-arterial injection.^38,39^ To our opinion, this is the first publication that presents such an innovative technology, which surpasses any of the current noninvasive visualization methods such as the vein viewer technology both by relevance and ease of use.

The main advantage of this MRA is that it provides the visualization of the individual arterial network in a noninvasive, radiation-free, and contrast-free. Although it initially requires a (small) effort of the patient, it remains a completely risk-free procedure. Moreover, a one-time MRA of the individual facial vascular anatomy may suffice for multiple years of repeated STF injections. A thorough literature search did not yield any publication discussing the consistency of the facial arterial anatomy with increasing age, apart from a recent study by Gombolevskiy et al claiming that the angular artery tends to lie deeper with increasing age, but only in some regions of the face.^40^ It seems quite difficult as well to study this hypothesis, as it would require a long follow-up period with repeated and potentially harmful computed tomography angiography (CTA) imaging. However, this new MRI sequence has the potential to provide an answer to this and many more anatomical brainteasers. As it requires no intravenous contrast, nor does it expose the patient to harmful radiation, the MRI may be repeated after a number of years (which may be the case in patients consulting their plastic surgeon for many years or even decades) and interesting anatomical discoveries regarding arterial location and depth may be discovered.

Depth info (based on the MRA data) may be added as well to the AR visualization by means of color coding or by visualizing actual depth information in millimeters. Knowledge of arterial depth seems much more relevant in an AR context than merely a descriptive summary based on anatomical studies. Indeed, seen the extremely tortuous course of most arteries in the face, the depth of an artery is constantly changing during its course. By providing both the arterial location and depth through AR, the injector is given very useful information while performing dermal filler injections to avoid potentially dreadful vascular complications. Additionally, accurate dynamic movement of the arteries may also be implemented in a future version. Within the current AR-tracking app, a dynamic movement of the arteries is already seen during facial animation: as the tracking reference points follow the changing facial contours, the visualized arteries move accordingly. However, currently a 1-to-1 movement of the arteries is seen, meaning the arteries move with the same dynamic range as do the facial contours. It is possible that superficial (subdermal) arteries are more dynamic than deeper (supraperiosteal) arteries. For that purpose, further investigation and development is needed.

### Study Limitations

A limitation of this study in regard to the analysis of anatomical variation is the sample size. However, even in this relatively limited group the extremely individual variability of the facial arterial anatomy (location and course of the DN, SO, and STr arteries) was already clearly shown (right and left side, in total 40 trajectories of each artery). The imaging and clinical utility was highlighted by the high interrater score and the satisfying image quality. These findings should be considered as a warning and proof that the anatomical variation—in this case in the region of the forehead—is significant and one can never be sure about the exact location of the facial arteries when only relying on the standard book anatomy. As more radiological centers might implement this newly developed MRI sequence, a bigger data pool may be extrapolated and analyzed. This in turn may allow us to further investigate the numerous anatomical variations (in location, course, and depth) of the most common facial arteries.

A second potential limitation of our study is that our IR-MRA 3D-TOF technique was not compared to CTA using contrast medium. Although all volunteers were healthy and without known allergies, we opted not to expose them to diagnostically unnecessary contrast media and radiation, both for medical and ethical reasons.

As there is no gold standard procedure that might prevent blindness when performing minimally invasive injectable procedures in the nasal or forehead region, we currently cannot evaluate the exact contribution of this new arterial visualization technique in the prevention of vascular filler complications. Neither can we organize any clinical study to test the assumptions made, based on ethical and moral considerations. However, we may presume that a thorough knowledge of the exact location and pathway of the main trunk of the DN, SO, and STr arteries is crucial to decrease the risk of puncturing an artery with a filler syringe. Moreover, DeLorenzi stated clearly that with respect to filler treatments, even with a good anatomical knowledge and a correct technique, there is still some “nonzero risk of vascular embolic events” (including highly skilled, experienced injectors).^41^ Awareness among the referring STF injecting physicians is growing and will also illustrate the added value of dedicated facial MRA. The same author also stated that avoidance of complications is still the best strategy.^41^

The setup of this study did not include a technical validation to evaluate the accuracy of the visualized arteries through AR. A follow-up study is planned to check the accurate positioning of the visualized arterial course by AR using ultrasound. This study will not only provide an answer to whether or not the AR visualization is accurate enough and can be trusted during filler injections, but also if the change in patient positioning—the patient is lying down during the MRI and is sitting up during the AR visualization and filler injection—may (slightly) affect the position of the facial arteries.

As our sample size is quite small (20 patients with 5 male and 15 female patients), we could not reach a high enough power to calculate potential significant differences in vessel visibility on MRI between genders and/or age groups. However, as the number of centers implementing this new MRI sequence increases, a larger database may be collected for future studies, which is very exciting. Interestingly enough, a recent study by Gombolevskiy et al did not find any significant difference in the course of the angular artery between both genders.^40^

Lastly, as we did not consider the many anastomoses between the different arteries of the face and the branches of the ophthalmic artery, nor very exceptional pathways (intracranial penetration during temporal STF injection), several other, albeit rare, scenarios are possible during which inadvertent canalization of a facial arterial branch may also lead to blindness.^20,42^

Further investigation to improve the visualization of the facial vessels is needed. Moreover, creating an awareness among patients and practitioners about the extreme variation of the individual vascular anatomy and of this potential life-altering condition is mandatory. Prevention of this grave and (mostly) irreversible complication remains the key and MRA may be part of it. Persuading every patient to perform an MRA in preparation of dermal filler injections seems like a mission impossible. However, seen the reasonable cost of a single MRI—around €250 in West-European countries—compared to the yearly expenses for repeated filler injections, paying the price for a noninvasive and risk-free imaging technique that may truly aid the injector in avoiding life-changing complications seems a fairly justifiable expense.

## CONCLUSIONS

This study represents the first comprehensive overview of the individual and very variable anatomy of the 3 main end branches (SO, STr, and DN artery) of the ophthalmic artery in a radiation-free, contrast-free, and noninvasive way. This risk-free imaging technique seems very useful in order to acquire accurate and patient-specific anatomical information prior to a STF injection. The combination of a MRA 3D-TOF MOTSA, followed by the AR visualization of the arterial anatomy may help to inject STF in a safer way and prevent blindness due to retrograde filler injection. Although the AR implementation was merely done in an experimental setting, this study provides nonetheless a glimpse in the future of modern medical technology.
